# Food Insecurity: A Barrier to Reproductive Justice Globally

**DOI:** 10.1080/19317611.2023.2201841

**Published:** 2023-04-21

**Authors:** Jasmine Fledderjohann, Sophie Patterson, Maureen Owino

**Affiliations:** a Department of Sociology, Lancaster University, Bailrigg Campus, Lancaster, UK; b Faculty of Health and Medicine, Lancaster University, Bailrigg Campus, Lancaster, UK; c Faculty of Health Sciences, Simon Fraser University, Burnaby, Canada; d Faculty of Environmental Studies and Urban Health, York University, Toronto, Canada

**Keywords:** Reproductive justice, food insecurity, human rights, structural violence, social determinants of health, reproductive choice, marginalization

## Abstract

***Objective:*** Reproductive Justice identifies three core reproductive rights for all people: (1) the right to not have a child; (2) the right to have a child; and (3) the right to parent children in safe and healthy environments. We aim to illustrate that food insecurity infringes upon on all three of these rights and so is a pressing issue for reproductive justice and for sexual and reproductive health more broadly.

***Methods:*** Using a phenomenological approach, we outline potential pathways between food insecurity and reproductive justice.

***Results:*** There are numerous potential pathways between food insecurity and reproductive justice, including entry into sexual relationships for material support, links to sexually transmitted infections and infertility, structural violence, prioritization and spending tradeoffs between food and other basic necessities, biological impacts of malnutrition, restricted reproductive choices, population control measures, and social stigma and exclusion. Marginalized people are disproportionately impacted by food insecurity and its consequences, with implications for sexual health and pleasure and for reproductive justice.

***Conclusions:*** Meaningful and equitable collaboration between people with lived experience of food insecurity, human rights and reproductive justice activists, and academics is critical to sensitively contextualize this work and mobilize broader social change.

## Introduction

Food insecurity is a situation in which people face difficulties accessing enough safe and nutritious food to support a healthy life (FAO et al., 2021). Key thinkers in the field of reproductive justice have listed food insecurity as one of many systemic social problems limiting the reproductive options available to marginalized people (Murray, 2021; Ross, 2017). However, the specific pathways through which food insecurity limits reproductive justice have not yet been detailed. We build on scholarship from reproductive justice activists, Black intersectional feminists, social scientists, and public health researchers to conceptualize food insecurity as a significant barrier to the realization of rights articulated in the reproductive justice framework. In the following sections, we briefly describe the reproductive justice framework and provide an overview of food insecurity as both a social problem and social determinant of overall health, including sexual health. We then outline in detail the myriad ways in which food insecurity threatens reproductive justice, and sexual and reproductive health more broadly. Because both food insecurity and reproductive justice are social justice issues affecting communities around the world, rather than focusing on one country or geographic region, we identify theoretical pathways with broad applicability across a range of contexts.

### Reproductive justice

Responding in part to the limitations of the pro-choice movement in the United States (US), a group of twelve Black women^1^ established the reproductive justice movement in 1994 as both a platform for action and an epistemological tool for centering the frequently devalued, trivialized, or ignored reproductive experiences and needs of repressed and marginalized people (Ross, 2017; Ross & Solinger, 2017). Intersectionality—a theory which highlights the compounding effect that experiencing multiple forms of systematic marginalization can have on all aspects of life (Crenshaw, 1991)—is central to reproductive justice. As an explicitly intersectional feminist framework, reproductive justice actively seeks to empower marginalized people both through theorization of systemic inequities to lift the veil on structural violence and, reciprocally, grassroots collective action to redress these inequities (Ross, 2017; Ross & Solinger, 2017).

While previous movements have often focused on a narrowly defined issue (e.g. the pro-choice movement), reproductive justice considers the full range of factors shaping the (lack of) childbearing and parenting choices and capabilities available to people, with focus on how institutional forces restrict options for marginalized people. Founding activist Loretta Ross and coauthor Rickie Solinger (2017) explain that, by incorporating “standpoint theory’, reproductive justice:

Helps us interrogate a host of injustices that may seem tangential to reproductive health, rights and justice – for example, gentrification, environmental degradation, incarceration, migration and militarization… [and] looks at how these issues intersect with each other and how, at various points of intersection, they affect the reproductive bodies of women and individuals. (pp. 72–73)

Reproductive inequities are understood not as simple cross-sectional social problems, but as systems of oppression with deep historical roots. Deconstructing systemic reproductive inequities requires examining legal structures that construct and maintain reproductive oppression of some people, and also examining how sociocultural norms about who can and “should” reproduce are shaped by, and help to perpetuate, historical inequities. Ross and Solinger (2017) observe that women who lack resources (whether money, food, etc.) are unable to fully enter the “marketplace of reproductive options’, encountering “choiceless choices,” which are made within a broader socio-political context that may further restrict reproductive autonomy. As a group disproportionately impacted by food insecurity (Pereira et al., 2021) and touched by a history of coerced sterilization and abortion (UNAIDS, 2020), people living with HIV are an important population-specific example. Harmful and distressing reproductive rights violations may also compromise the right to (sexual and general) health by creating barriers to accessing HIV care and engaging with antiretroviral therapy (ART) (Mavundla et al., 2022). In 2020, UNAIDS released a statement acknowledging the importance of prioritizing reproductive health of all women, regardless of HIV status (UNAIDS, 2020); nonetheless, people living with HIV continue to face particularly severe and intersecting inequities that structurally limit their sexual and reproductive rights.

Reproductive justice considers how multiple oppressions intersect to impair the realization of human rights for marginalized people. As Ross and Solinger (2017) clarify, reproductive justice “begins with the proposition that while every human being has the same human rights, not everyone is oppressed in the same way, or at the same time, or by the same forces” (p. 72). Learning from human rights claims made by women in the Global South (Ross, 2017), reproductive justice draws on global human rights standards and treaties, viewed intersectionally. This conscious choice to use global human rights as a philosophical and legal framework is powerful because it establishes the international scope of reproductive justice, rather than restricting the focus to specificities of reproductive oppression seen in the US.

Reproductive Justice focuses on three core rights: The right to have a child; the right to not have a child; and the right to parent children with dignity in safe and healthy environments (Ross, 2017; Ross & Solinger, 2017). Importantly, each of these rights clearly connects back to sexual health as part of overall health. These rights also highlight that, though clearly essential, equitable access to high-quality healthcare is but one aspect of reproductive justice. One cannot choose if, when, and how often to reproduce (defining reproductive health choices according to WHO, 2013) without access to both reproductive healthcare, e.g. contraception and abortion care, and the means to meet the basic needs of oneself and one’s (potential) family safely and with dignity. Inequities embedded in the broad social, political, economic, and physical settings in which we live are not distinct from sexual and reproductive health, but central to it (Ross & Solinger, 2017). In this paper, we focus on food insecurity because it remains an important but under-theorized barrier to reproductive justice.

### Food insecurity

Food insecurity is a “wicked problem”: A challenging global public health issue with multiple complex causes and a lack of simple solutions (Walls, 2018). It is correlated with, but distinct from, both poverty and malnutrition. In its milder form, food insecurity may entail eating less preferred foods and worrying about food access, while more severe forms of food insecurity may also include skipping meals or going without food entirely. It is a multidimensional concept, involving availability of, access to, and utilization of food, as well as (in)stability of each of these dimensions over time (Webb et al., 2006). Food insecurity may be experienced across one or multiple dimension(s), and may vary over time. An individual working a low-wage job with unsteady hours, for example, may usually find enough safe and healthy food available locally to grow or purchase, but sometimes reduced financial resources may limit their access to sufficient food despite its local availability.

The experience of food insecurity is shaped by a multitude of interconnected actors and processes in the food system. The food system entails not only macro-level factors, such as the health and stability of the ecosystem, large-scale agricultural practices, international trade policy and tariffs, systems for processing and distributing food, food culture, and social policy, but also micro-level factors including socioeconomic status, geospatial patterns of daily life, and individual food preferences (Clair et al., 2021; Nguyen, 2018). Although Covid-19 has drawn international attention to and exacerbated food insecurity, it is not a pandemic-specific issue; rather, food insecurity is a longstanding problem rooted in large-scale, structural inequities (Clair et al., 2021). Globally, food insecurity is likely to be compounded in coming years due to food system instability driven by structural problems, including systematic divestment in social protection, political instability, and the climate crisis (Clair et al., 2021; FAO et al., 2021; Pérez-Escamilla, 2017).

Food insecurity is a social determinant of health. A social determinants perspective recognizes that the conditions in which we are born, live, work, and age have the greatest impact on our health and well-being (Dahlgren & Whitehead, 2021; Marmot, 2005). Gradients in social and community networks (influencing food culture); living and working conditions (including food availability and consumption), and broader socio-economic, cultural, political, and environmental contexts (including geospatial environments, food policy, and social protection systems) shape opportunities for health and well-being, including sexual and reproductive health, in ways that construct layers of disadvantage among marginalized groups.

The Universal Declaration of Human Rights asserts the right to food as essential for all people (United Nations, 1948). Subsequent international human rights documents have clarified and, in some places (e.g. India), codified into law, the right to food. The UN Economic and Social Council (1999) clarified that the right to food is “inseparable from social justice,” asserting states must take progressive action to respect, protect, and fulfill the right to food for *all* people under the state’s jurisdiction. Simply not infringing upon the right to food is insufficient; states must actively safeguard this right and ensure it is being met for all. Nonetheless, substantial inequities within and between communities remain. As with reproductive justice, the experience of food insecurity is shaped by intersecting inequalities. Groups facing the greatest risk of food insecurity vary across contexts, but extant research has identified low-income households, racially marginalized people, female-headed households, people with disabilities, transgender and gender nonconforming people, unemployed people, pensioners, refugees and undocumented immigrants, people living with HIV, people who experience gender-based violence, and people without homes or precariously housed individuals as those experiencing the highest prevalence and most severe forms of food insecurity (Brucker & Coleman-Jensen, 2017; Clair et al., 2020; Kakota et al., 2015; Loopstra et al., 2019; Magaña-Lemus et al., 2016; Pereira et al., 2021; Russomanno et al., 2019; Smith et al., 2017). Consistent with the fundamental commitment to community empowerment within the reproductive justice framework, the UN Food and Agriculture Organization (FAO et al., 2021) advocates for empowering marginalized groups with limited access to resources, power, or influence through provision of resources, technology, and education to tackle food insecurity.

## Food insecurity: a barrier to reproductive justice

We examine the social science and public health literature on food insecurity through the lens of reproductive justice. Social epidemiology has significantly advanced our understanding of social gradients in health, but individual agency and structural inequities that constrain choices for some have historically been neglected in this literature (Frohlich & Abel, 2014). Reproductive justice, in contrast, takes an intersectional view, considering both individual choice and difference, and interrogating how structural barriers constrain choice, and for whom.

Food insecurity is often conceptualized and measured at the household level,^2^ leaving gender as a frequently neglected component of food insecurity research (Broussard, 2019). However, there is increasing recognition of the importance of addressing food insecurity through a gender lens (Visser & Wangu, 2021). FAO et al. (2021) recently found the global prevalence of moderate or severe food insecurity was 6% higher for women than for men, with this gap growing to 10% during the Covid-19 pandemic. In some countries, the gender gap was as high as 19% before the pandemic (Broussard, 2019). The dominant role of women as caregivers within the family and their involvement in global food production cements their influence over food security (Visser & Wangu, 2021). Similarly, women contribute to early infant nutrition through their diet in the pre-conception and antenatal period and through breastfeeding postnatally (Fledderjohann et al., 2016; Stephenson et al., 2018). However, restrictive social norms, maternal responsibilities, and other gender-based barriers can limit women’s control over resources, equal participation in household decision-making related to food, and opportunities for involvement in agriculture.

An intersectional approach recognizes women are not a homogenous group, and food insecurity is experienced differently based on interconnected forms of oppression and marginalization, driven by *intersecting* social identities that shape access to power (Crenshaw, 1991; Ross & Solinger, 2017). For example, qualitative research from Canada illustrates interconnected and overlapping identities that shape risks and experiences of food insecurity among women living with HIV, including Indigenous ancestry, racial marginalization, migrant status, gender, and sexual orientation (Sernick et al., 2022). Women reporting intersecting marginalized social positions, including racially marginalized women living in poverty, describe a simultaneous lack of culturally-appropriate, trauma-aware service provision and suboptimal social support services, limiting opportunities and empowerment to achieve food security. Social positions that increase food insecurity risks may similarly drive reproductive oppression and may negatively impact sexual health.

Because reproductive justice compels us to consider gender and how it intersects with other identities and forms of marginalization, it naturally requires us to consider dynamics at the sub-household level. Likewise, because safe and healthy environments are a core right, reproductive justice highlights children and young people’s experiences, which have been comparatively neglected in food insecurity research (Hadley et al., 2009). A reproductive justice approach to food insecurity, then, facilitates a more nuanced understanding of food insecurity. We argue food insecurity threatens rights captured under the reproductive justice framework in a myriad of ways, with impacts disproportionately felt by (multiply) marginalized women.

### The right to not have a child

We hypothesize three key pathways through which food insecurity may threaten the right to not have a child: (1) entry into sexual relationships for material support, (2) restricted sexual autonomy within partnerships, and (3) tradeoffs between expenditures on food and expenditures on reproductive healthcare.

In the first pathway, studies in both Global Majority and Global Minority countries have shown food insecure women may utilize transactional or commercial sex to feed themselves and their children (Govender et al., 2022; Weiser et al., 2011), especially (but not exclusively) in settings where women have limited property, inheritance, and land ownership rights (Amin, 2015). Young people may also face unique barriers to accessing employment and social protection programs, leaving them to seek out creative means (which may be stigmatized or criminalized) of meeting their food needs (Govender et al., 2022). As Laverty’s (2019) ethnographic study in England showed, food insecure young people may share food through informal networks. However, because of gendered eating norms and expectations for girls to perform caring through food sharing, girls may be less able than boys to benefit from peer networks to acquire food. Perhaps linked to this gender inequality, some girls turned to romantic relationships to meet their food needs, with notable age gaps between adolescents and their adult partners. Research with adolescents and young adults in other settings (e.g. South Africa, see Fielding-Miller et al., 2015; the US, see Mmari et al., 2019) likewise shows that food insecure girls and women sometimes rely on sexual partnerships (ranging from sex work to long-term romantic relationships) to obtain food and/or money, whereas boys and men were more likely to rely on activities including theft and selling drugs. Where criminalized activities to obtain food lead to incarceration, difficulty accessing abortion care poses a further infringement on the right to not have a child (Hayes et al., 2020). The stigma associated with transactional sex and the hypervisibility of women’s sexuality (particularly for racially marginalized women) has also been found to contribute ‘to discomfort in their sexual bodies’, diminishing sexual pleasure and, for some, creating a barrier to accessing sexual and reproductive healthcare (Chauke & Segalo, 2021).

Although mentioned by only one participant, Mmari et al.'s (2019) study in the US also found evidence that food insecure men and transgender women may engage in transactional or commercial sex to obtain basic necessities. While food insecure young people of all genders engage in a range of strategies to acquire food, extant literature shows that gender asymmetries place cisgender girls and women at disproportionate risk of unwanted pregnancy and other sexual and reproductive health issues. It is difficult to ascertain whether the infrequent mention of transgender people’s strategies to address food insecurity in the literature reflects differences in utilization of transactional or commercial sex (and associated pregnancy risks) or invisibilization of their experiences.

Turning to the second pathway, underpinned by pervasive gender inequalities and asymmetrical power dynamics, food insecurity may compromise reproductive autonomy within sexual partnerships (Amin, 2015). Research from Global Majority countries (Diamond-Smith et al., 2017; Smith et al., 2021) has found moderate and severe food insecurity was associated with a lower likelihood of using family planning methods, even after controlling for sociodemographics. Food insecure women face a greater risk of sexual violence (Weiser et al., 2011), and may be compelled to remain in violent relationships with limited sexual agency (Amin, 2015). An association between food insecurity and intimate partner violence (IPV) has been reported in the US (Breiding et al., 2017; Ricks et al., 2016) and other global settings (Awungafac et al., 2021; Hatcher et al., 2019). Indirect pathways proposed between food insecurity and IPV include increased relationship conflict and stress, mental distress, and reduced household well-being (Buller et al., 2016; Hatcher et al., 2019). IPV may distort the environment within which people navigate reproductive choices, compromising their ability to negotiate condom use within sexual partnerships, resulting in a higher prevalence of unprotected sex and unintended pregnancies (Lewis, 2018). In the UK, women exposed to IPV were more than twice as likely to have consulted a doctor for emergency contraception compared to those unexposed (Jackson et al., 2019).

Our third pathway considers tradeoffs between expenditures on food and reproductive healthcare. Research from the US showed more than half of women who didn’t utilize insurance to pay for an abortion reported difficulty paying for the procedure, of whom one-in-six cut spending on food to save for associated costs (Jones et al., 2013). Similarly, in Arizona nearly one-in-five women reported cutting spending on food to afford abortion costs, rising to more than one-quarter when looking at women below the poverty line (Karasek et al., 2016). What these studies of women seeking abortions cannot capture are the experiences of food insecure women who wanted an abortion but could not obtain one. One mixed-methods study among women seeking prenatal appointments in the US showed food insecurity was significantly associated with having considered an abortion, but was also associated with higher odds of reporting a policy-related barrier (most commonly bans on public funding for abortion) to obtaining an abortion (Roberts et al., 2020). Where geographic accessibility of abortion services is limited, and particularly where policies such as mandatory waiting periods increase the amount of time required to obtain an abortion, the resources required can be especially burdensome for women living in remote areas (Ely et al., 2019).

Tradeoffs between food and abortion care costs may impede the right to not have a child. Such structural violence can simultaneously limit other rights (Clair et al., 2021). While freely available abortion care is a necessary condition for reproductive justice, free care alone is insufficient for ensuring reproductive justice. As Coast et al. (2021) explain, abortion costs include the entire care-seeking trajectory, not only costs at the point of care. For example, where strict gestational limits on legal abortion mean women must act quickly to obtain an abortion, the time needed to identify costs of the full care-seeking trajectory and obtain the necessary financial resources may result in women surpassing gestational limits and being denied an abortion (Upadhyay et al., 2014). People in precarious employment may struggle to negotiate time off work or even risk unemployment for missed work, particularly if encountering geographical or structural barriers to attendance (e.g. waiting periods requiring multiple appointments). Transportation costs, lost wages, and childcare costs are all financial barriers to obtaining an abortion above and beyond the cost of the procedure itself (Jones et al., 2013), which are likely to disproportionately burden women experiencing food insecurity.

Multiply marginalized people may face further, unique barriers that restrict their ability to obtain an abortion, even where abortion is available with no cost at the point of care. For instance, as activist and reproductive health service provider Chabot (2021) highlights, the Covid-19 pandemic in Canada reduced service provision through hospitals and clinics while simultaneously introducing barriers, such as border closures and cancelation of public transport routes. Such barriers disproportionately impacted marginalized people, especially those with precarious immigration status who could not, for example, cross into the US to access care. These new barriers made the competition between food and abortion care-seeking trajectory costs even greater. Similar compounding of such cost tradeoffs may also apply where abortion is illegal and pregnant people must risk great expense and danger to themselves to obtain needed care. Meanwhile, even where abortion is legal and accessible, poor and racially marginalized mothers’ parenting is under constant social scrutiny, including their ability to consistently provide “healthy” food, and enough of it (Chabot, 2021; Fielding-Singh, 2021). For these mothers, the stigma of risking opportunity costs to obtain an abortion (e.g. risking unemployment and potential food insecurity) may add further strain to an already difficult decision. As sociologist Hays (1996) explained of US mothers:

…middle- and upper-class whites are not only liable to maintain the economic benefits connected to their class and race but also are likely to gain the advantage of certain social legitimacy for their economically and culturally privileged position. (p. 164)

### The right to have a child

Food insecurity may restrict realization of the right to have a child in a multitude of ways, including through impacts on one’s biological capacity to have a child; postponement or termination of pregnancy due to constrained reproductive choices; and systems of structural violence that restrict the fertility of food insecure people.

A robust, global literature has established that food insecurity is associated with caloric and micronutrient deficiencies, anthropometric failure, anemia, diabetes, and other markers of malnutrition (Gucciardi et al., 2014; Johnson et al., 2018; Moradi et al., 2018). Malnutrition has been linked to subfecundity (Panth et al., 2018); operating through nutritional risks, food insecurity (particularly severe, chronic food insecurity) may reduce one’s ability to conceive. Because maternal nutrition is vital for fetal development (Stephenson et al., 2018), food insecurity is, throughout pregnancy, also a barrier to the right to have a child. Moreover, food insecurity may create a choiceless choice in the form of a tradeoff between food and reproductive healthcare costs (including transport and opportunity costs), with implications for general sexual and reproductive health and antenatal checkups (Weiser et al., 2011). Stress over affording food and other basic necessities may also influence postponement of fertility and/or the decision to terminate a pregnancy. A recent study in Tanzania, for example, identified a preference among food insecure women to delay or avoid pregnancy (DiClemente et al., 2021).

As outlined above, some food insecure people may utilize transactional or commercial sex to obtain food and financial resources (see e.g., Govender et al., 2022). Power asymmetries and limited negotiating power around condom use in some relationships of this nature increase the risk of exposure to STIs (Amin, 2015), which, if untreated, increase the risk of infertility (Tsevat et al., 2017). Criminalized strategies for obtaining food also place some food insecure people at risk of incarceration. As Hayes et al. (2020) explain, focusing on the US, incarceration restricts one’s ability to become pregnant, and can also result in forced sterilization. The low quality of prenatal care in many prisons restricts access to the healthcare needed to sustain healthy pregnancies, and often also results in delivery in inhumane conditions (e.g. shackling during labor, separation from their newborns within <24 h of birth). And, as Hayes et al. (2020), Roberts (1997), and many other intersectional feminists have highlighted, in the US the criminalization of poverty and the so-called “War on Drugs” have disproportionately targeted multiply marginalized people, particularly Black women, making this a deeply racially and socioeconomically stratified infringement of the right to have a child.

Structural reproductive coercion can be manifested at the state level through policies that shape the environment within which reproductive decision-making occurs. For example, the introduction of the UK’s two-child limit policy in 2017 limited the financial support available to low-resource families with more than two children, except in limited circumstances (Clair et al., 2021). This policy was criticized as selectively altering the reproductive decision-making context of families relying on welfare support, signaling that having more than two children was a class privilege. In 2020, a British Pregnancy Advisory Service (BPAS, 2020) survey of 240 women with two or more children who had decided to terminate a pregnancy showed more than half of women who were aware of the policy and in receipt of social support indicated the policy influenced their decision. Meanwhile, data on food insecurity from 2019 show that around one-quarter of UK households receiving income support were food insecure, and 43% on Universal Credit were food insecure (Clair et al., 2021). At the same time, lone-adult households with children faced the highest risk of food insecurity out of any group, with over one-quarter of lone-adult households with one or two children reporting moderate or severe food insecurity, and with 41% of those with three or more children reporting food insecurity. While it’s not possible to make a causal claim from these descriptive trends, taken together this evidence suggests that state policies are not meeting the right to food, thereby establishing the conditions for choiceless choices and selectively restricting reproductive autonomy.

Similarly, international development rhetoric and many associated family planning programs seek to reduce the “high” fertility of poor women in Global Majority countries in the name of reducing food insecurity through population control (Senderowicz & Higgins, 2020). In this globally stratified system of structural violence, some programs pressure or outright coerce people who can become pregnant into utilizing contraception, particularly long-acting reversible contraceptives (LARC).^3^ Senderowicz and Higgins argue that programs which advocate fertility reduction and LARC as a panacea for food insecurity exploit women’s bodies in the name of achieving societal goals, unacceptably limiting their reproductive and sexual autonomy. We add to this that the world’s wealthiest people and countries have long been the cause of the greatest environmental destruction (Klein, 2020). Meanwhile, the climate crisis is directly responsible for a large and growing fraction of food insecurity globally. If the justification for population control is to address food insecurity, the choice to target marginalized people who can become pregnant in Global Majority countries is quixotic (at best). More importantly from a reproductive justice perspective, a poor person in a Global Majority country has no less right to have a child than does, for example, a wealthy white cisgender man in a Global Minority country. We do not propose shifting reproductive coercion to focus on the bodies of wealthy white cisgender men in Global Minority countries. Rather, globally marginalized people should not be expected to solve large-scale social problems by foregoing their right to have a(nother) child. Globally, marginalized people currently experience a double-jeopardy of structural violence in the form of reproductive coercion and climate crisis risks, including food insecurity. This is deeply unjust.

### The right to parent children with dignity in safe and healthy environments

From its impacts on pregnancy experiences to increasing risks of malnutrition, diminished cognitive development, and poorer socioemotional well-being, food insecurity shapes the environments in which children are born and grow. Food insecurity can force parents to make choiceless choices in the form of difficult tradeoffs to meet their children’s basic needs. It is a clear threat to the right to parent children with dignity in safe and healthy environments.

From the earliest stages of child development, food insecurity can shape future health and well-being. Food insecurity during pregnancy can compromise neonatal health outcomes, increasing the risk of birth "defects" and neonatal death, and is associated with early discontinuation of breastfeeding, with implications for future nutrition and well-being (Augusto et al., 2020). A study from Haiti also identified food insecurity as a risk for preterm births (Richterman et al., 2020). In a recent commentary, Laurenzi et al. (2020) underlined the importance of examining intersections between food insecurity, maternal mental health, and IPV to support optimal maternal and child health outcomes, acknowledging that exposures may interact to heighten the risk environment within which children are conceived and raised.

Food is essential not only for children’s growth and survival, but also for psychosocial well-being. Literature from around the globe has shown an association between food insecurity and anthropometric “failure,” micronutrient deficiencies, anemia, reduced immunity, risk of chronic conditions, such as diabetes, and even death (FAO et al., 2021; Gucciardi et al., 2014; Moradi et al., 2018; Thomas et al., 2019). Nationally representative data from the US revealed an association between household food insecurity and suboptimal general child health, acute and chronic health conditions, and non-adherence to recommended healthcare practices (Thomas et al., 2019). Exposure to food insecurity during childhood and adolescence, particularly persistent food insecurity, can have lasting impacts across the lifecourse, not only for nutrition and physical health, but also for socioemotional well-being, sexual behavior, cognitive development, and school achievement. After accounting for sociodemographics, children and young people who experience food insecurity face greater difficulties concentrating, perform less well on tests of learning and cognitive ability than their peers, are more likely to drop out of school, and are more at risk of experiencing bullying and, in adolescence, engaging in substance use and having unprotected sex (Argaw et al., 2023; Aurino et al., 2019; Jyoti et al., 2005; Martinez et al., 2020; Paquin et al., 2021; Smith et al., 2021).

A safe and healthy environment must not only be physically safe, but also must promote socioemotional and mental well-being (Ross & Solinger, 2017). For both parents and children, food insecurity is a source of stress, shame, social stigma, social exclusion, and mental distress (Fielding-Miller et al., 2015; Fielding-Singh, 2021; Garthwaite, 2016; Martinez et al., 2020). Purdam et al. (2016) highlighted the important role of food in creating a “sense of home” in the context of family life in the UK; food insecurity can negatively impact self-worth and contribute to social isolation due to restricted ability to host social gatherings centered around food.

In another pathway between food insecurity and safe and healthy environments, food insecure people who utilize criminalized strategies for obtaining food face the risk of incarceration. In turn, incarceration of adults (Cox & Wallace, 2016), and pregnant people or their partners (Testa & Fahmy, 2021) is linked to an increased risk of food insecurity. Incarceration directly infringes upon the right to parent children with dignity in safe and healthy environments. In the US, for example, this infringement occurs by separating parents from their children and by limiting access to employment and to safe and affordable housing following incarceration (Hayes et al., 2020). This infringement is highly gendered—where children are placed in foster care during incarceration, mothers are more likely to lose parental rights than fathers.

Even where children are not separated from parents, however, food insecurity can still force parents to make choiceless choices in the form of tradeoffs between basic necessities. Food insecurity may push households to live in poorer quality housing, to live in less safe neighborhoods, and/or to forgo heating or cooling even in extreme temperatures to afford food (Bhattacharya et al., 2003; Clair et al., 2020; Garthwaite, 2016), leaving families facing the negative health and psychosocial consequences of such conditions (Clair, 2019; Clair et al., 2021). Extant work has documented a similar tradeoff between food insecurity and healthcare, including not only the costs of necessary procedures, medical visits, prescriptions, etc., but also ancillary costs, such as energy to refrigerate prescriptions where needed (Berkowitz et al., 2014; Clair et al., 2021).

And, as Fielding-Singh's (2021) ethnographic study of Californian families shows, food insecurity can force choiceless choices between tending to children’s physical *vs.* socioemotional well-being. Challenging the public health literature on food deserts and nutritional health disparities, Fielding-Singh explains generally all mothers^4^ cared about good childhood nutrition, and the vast majority were able to access spaces where healthy food could be purchased. However, low-income mothers were more inclined to say “yes” to children’s junk food requests, in part because, in a parenting context requiring them to regularly say “no” to their children, food was a small way they could say “yes” (Fielding-Singh, 2021). This act of saying “yes” was important for the socioemotional well-being of both mothers and children. Additionally, though sometimes unhealthy, children were less likely to waste (financially precious) food and potentially go hungry when food met their preferences—a particular concern for mothers who had experienced food insecurity. Conversely, mothers with greater financial resources often took pride in saying “no” to junk food, feeling this was a way to signal that they were “good” mothers who make healthy choices. Concerns about food waste were not a factor for these mothers. However, high-income mothers also explicitly mentioned junk food they exceptionally allowed to show that they were not overly privileged nor elitist. As Fielding-Singh explains, however, comfort to discuss these choices without fearing stigmatization is itself a marker of privilege. Mothers who experience both socioeconomic security and white privilege epitomize (US) sociocultural notions of what makes a “good” mother, and so can afford to call attention to typically stigmatized choices; for racially and/or economically marginalized mothers, however, the same action is stigmatized as a failure to provide adequate nutrition for growing children.

Reproductive justice conceptualizes environments as sociocultural and political entities, not just localized geophysical spaces. The food system is part of these environments. One’s position in that system dramatically shapes who accesses what food, when, and how. This system includes a food industry that aggressively markets sugar-laden, highly processed foods to children, young people, and their parents (Fielding-Singh, 2021). This increases the desirability of such products, and, as Fielding-Singh’s work powerfully demonstrates, leaves low-income parents with the choiceless choice of tending to their children’s socioemotional well-being by purchasing junk food or tending to their physical health but leaving them feeling deprived and socially excluded. And, as social constructions of race and class interact to create a double-standard of food caring work for mothers, nutritional inequities are also felt by their children.

## Discussion

This paper presents a theoretical perspective that is underexamined in the global literature. We have outlined some of the pathways through which food insecurity may infringe on the right to have a child, the right to not have a child, and the right to parent children with dignity in safe and healthy environments. Some of these pathways include entry into sexual partnerships for material support, limitations on sexual autonomy in relationships, prioritization and spending tradeoffs between food and other basic necessities, biological impacts of malnutrition, restricted reproductive choices, structurally violent population control measures, and social stigma and exclusion. We have highlighted but a few of the many ways that marginalized people are disproportionately impacted by food insecurity and its consequences for reproductive justice. The links between food insecurity and reproductive justice detailed here demonstrate an urgent need for further scholarship to document and theorize food insecurity as a source of reproductive oppression through the lens of reproductive justice. Collaboration between people with lived experience of food insecurity, human rights and reproductive justice activists, and academics is critical to sensitively contextualize this work and mobilize broader social change.

The experience of food insecurity is not siloed; it cuts across all three rights. In many instances, it was difficult to decide under which heading to put some experiences because different individuals will experience the same phenomenon uniquely; universality of experience cannot be assumed. For example, we suggested that transactional sex to acquire food infringes upon the right not to have a child because of extant research highlighting that this situation can create power imbalances, with implications for the ability to prevent pregnancy and refuse sex. However, to assume that limited negotiation power is universal in transactional sex is disempowering and reductionist. Therefore, while we have aimed to represent key ways in which food insecurity is a barrier to reproductive justice for illustrative purposes, this should not be taken as a comprehensive accounting. Nor do we claim that a particular aspect of food insecurity impacting one right does not also have implications for other rights, within the reproductive justice framework and beyond. For instance, while not the central focus of this manuscript, the literature discussed suggests that the right to enjoy safe and healthy sexual relationships may be jeopardized by experience of food insecurity (see e.g., Chauke & Segalo, 2021). Sexual pleasure is a key determinant of general, mental, and sexual well-being, and the importance of a pleasure-based approach to sexual health and sexual rights has been highlighted in recent work (Ford et al., 2021). In principle, if one’s basic needs are not met, it may be difficult to pursue safe and pleasurable sex. Importantly, however, empirical evidence on this association is limited. The relationship between food insecurity and sexual pleasure, health, and rights across the lifecourse merits further scholarship.

In a similar vein, food insecurity often does not occur in isolation, but is linked to other forms of insecurity and precarity, such as water insecurity and housing insecurity (see e.g., Brewis et al., 2020; Clair et al., 2020). We have focused narrowly on food insecurity here to facilitate an in-depth exploration of the many links between food insecurity and reproductive justice. Future research should consider how other forms of insecurity and precarity are relevant to reproductive justice, both because they are often linked to food insecurity, which we have in turn shown is intimately linked to reproductive justice, and because they independently have important implications for the right to have a child, not have a child, and parent children in safe and healthy environments (Brewis et al., 2020; Clair, 2019; Fledderjohann et al., 2015; Misra, 2014; Ross & Solinger, 2017).

Importantly, while we draw on scientific evidence to highlight the importance of nutrition throughout the life course, including during pregnancy, we in no way endorse the harmful notion that autonomy (e.g. choices about food consumption) should be restricted because nutrition during pregnancy and chestfeeding are scientifically linked to fetal and infant development. A powerful feminist literature has highlighted the multitudinous ways in which scientific discourses have been problematically utilized to restrict reproductive autonomy and assign blame to people who can become pregnant and to mothers in the name of safeguarding fetal and infant health (see e.g., Cescutti-Butler et al., 2019; MacKendrick & Cairns, 2019; Parker, 2020; Wall, 2001). Instead, we argue that food insecurity restricts reproductive autonomy by limiting options for food-related decision-making, resulting in choiceless choices. In other words, we aim to unveil the structural forces that constrain choice, not to pass judgment on the choices individuals make.

The theoretical framing presented within this paper was purposely geographically and temporally broad, based on the recognition that both food insecurity and reproductive justice are human rights issues that cut across time and place. However, further scholarship should examine this issue within specific contexts, circumstances, and population groups, particularly those disproportionately or uniquely affected by issues related to food insecurity and reproductive justice, and/or underrepresented in the literature. For example, experiences of transgender people are lacking in this field of scholarship, and future enquiry is needed. Indeed, where we use the language of “women” throughout the manuscript, this reflects the word’s use in statistics on food insecurity and in the broader literature, typically without clarification on who is included or excluded in research. It is frequently impossible to know whether transgender and gender nonconforming people were included in research from descriptions of study samples. Similarly, there is a critical need to represent the perspectives of Indigenous people, who navigate reproductive decision-making on a historical backdrop of human rights, cultural, territorial, and food sovereignty violations (Richmond et al., 2020). And, as conflict continues to be a major driver of food insecurity, undernourishment, and famine in a growing number of regions around the world (FAO et al., 2021), understanding the intersection of food insecurity and reproductive justice for people living in and displaced from these regions will be critical. Key to this work will be meaningful involvement of affected populations to ensure research is grounded in lived experience and empowers the population it seeks to represent, in keeping with principles of reproductive justice. Future empirical work that draws explicit attention to links between food insecurity and reproductive justice while centering the voices and experiences of people with lived experience would be valuable not only for advancing academic literature but also, importantly, for serving activists who seek to engage with policymakers to inform evidence-based policymaking.

We have drawn on scholarship from activists and academics, but we also write from perspectives shaped by our own social positions and experiences. JF is a sociologist by training, with expertise in social inequalities, food insecurity, and health disparities. She is a white cisgender woman with lived experience of childhood poverty, violence, and their sequelae. SP is a clinical academic, with expertise in public health and sexual and reproductive health and rights. She is a white cisgender woman with lived experience of motherhood and pregnancy loss. MO is an activist scholar currently undertaking her PhD at York University in Toronto, Canada. She is a Black woman, Immigrant and person with lived experience of poverty, food insecurity and violence. Additionally she has lived experience of motherhood, coerced and forced abortion among other things. We have drawn on our professional expertise and personal lived experiences in the drafting of this work, but also note that other diverse voices are needed to further nuance this discussion.

This paper highlights a need to address gender inequalities that continue to power food insecurity and oppression of reproductive justice on a global scale. Gender equity and empowerment are critical to food system transformation and achieving food security globally. A central, unifying aim of the UN Sustainable Development Goals is to “realize the human rights of all and to achieve gender equality and the empowerment of all women and girls” (UNGA, 2015). As such, achieving the goal to “End hunger, achieve food security and improved nutrition and promote sustainable agriculture” by 2030 necessitates a central focus on gender equity. There is a global recognition of the importance of food security and women’s rights in advancing global health and sustainable development. A recent analysis of synergies across international sustainability and development agendas identified gender equality and food security as the two themes mentioned consistently across all four agendas (Bowen et al., 2021). We call on policymakers and academics to learn from reproductive justice activists and view these connections through an intersectional reproductive justice lens.

## Data Availability

Data sharing not applicable to this article as no datasets were generated or analyzed during the current study.

## Notes

1 Collectively, Women of African Descent for Reproductive Justice.

2 Particularly in survey research prior to the advent in 2014 of FAO’s Food Insecurity Experience Scale (FIES) module for measuring individual-level food insecurity.

3 While there have also been disturbing efforts to push LARC on poor and racially marginalized people in Global Minority countries, such efforts tend to utilize language rooted in poverty and personal responsibility narratives. This rhetoric is also deeply stigmatizing and extremely harmful, but does not usually frame this reproductive coercion as a means of addressing the structural problem of food insecurity and so is beyond our scope here.

4 Fielding-Singh observed several families and conducted interviews with many more. While fathers were present in many of these families, and were the primary caregivers in some, she focuses mainly on mothers both because they were disproportionately tasked with food-related caregiving and, relatedly, because prevailing sociocultural norms dictate that providing nourishment is an essential part of “good” mothering, from consuming healthy foods during pregnancy to breastfeeding to making and monitoring food choices throughout childhood and adolescence.
